# Precise design strategies of nanomedicine for improving cancer therapeutic efficacy using subcellular targeting

**DOI:** 10.1038/s41392-020-00342-0

**Published:** 2020-11-06

**Authors:** Xianglei Fu, Yanbin Shi, Tongtong Qi, Shengnan Qiu, Yi Huang, Xiaogang Zhao, Qifeng Sun, Guimei Lin

**Affiliations:** 1 Department of Pharmaceutics, Key Laboratory of Chemical Biology (Ministry of Education), School of Pharmaceutical Sciences, Cheeloo College of Medicine, Shandong University, Jinan, 250012 Shandong China; 2 School of Mechanical and Automotive Engineering, Qilu University of Technology (Shandong Academy of Sciences), Jinan, 250353 Shandong China; 3 The Second Hospital, Cheeloo College of Medicine, Shandong University, Jinan, 250033 Shandong China

**Keywords:** Drug delivery, Drug development

## Abstract

Therapeutic efficacy against cancer relies heavily on the ability of the therapeutic agents to reach their final targets. The optimal targets of most cancer therapeutic agents are usually biological macromolecules at the subcellular level, which play a key role in carcinogenesis. Therefore, to improve the therapeutic efficiency of drugs, researchers need to focus on delivering not only the therapeutic agents to the target tissues and cells but also the drugs to the relevant subcellular structures. In this review, we discuss the most recent construction strategies and release patterns of various cancer cell subcellular-targeting nanoformulations, aiming at providing guidance in the overall design of precise nanomedicine. Additionally, future challenges and potential perspectives are illustrated in the hope of enhancing anticancer efficacy and accelerating the translational progress of precise nanomedicine.

## Introduction

Nanoparticle-based drug delivery systems (NDDSs) are extensively employed in the therapy, diagnosis, and imaging of cancer due to their characteristics of high cancer-targeting efficacy, low toxicity, and controlled release properties.^1^ An efficient drug delivery system must avoid the clearance of the reticuloendothelial system, penetrate across blood vessel walls and be enriched at cancer sites to exert their pharmacological effects.^2^ For this purpose, an ever-increasing number of preclinical studies have reported a large number of engineered nanoformulations with unique physical and chemical properties, with the goal of delivering chemotherapeutic agents, photosensitizers, genes, and other biomolecules to cancer cells in specific and efficient manners.^3^ However, due to the problems of multidrug resistance (MDR), high variability, and poor patient prognosis, NDDSs have still faced tremendous challenges. It is therefore necessary when designing new treatment strategies to study in-depth the pathogenesis of cancer.

**Fig. 1:**
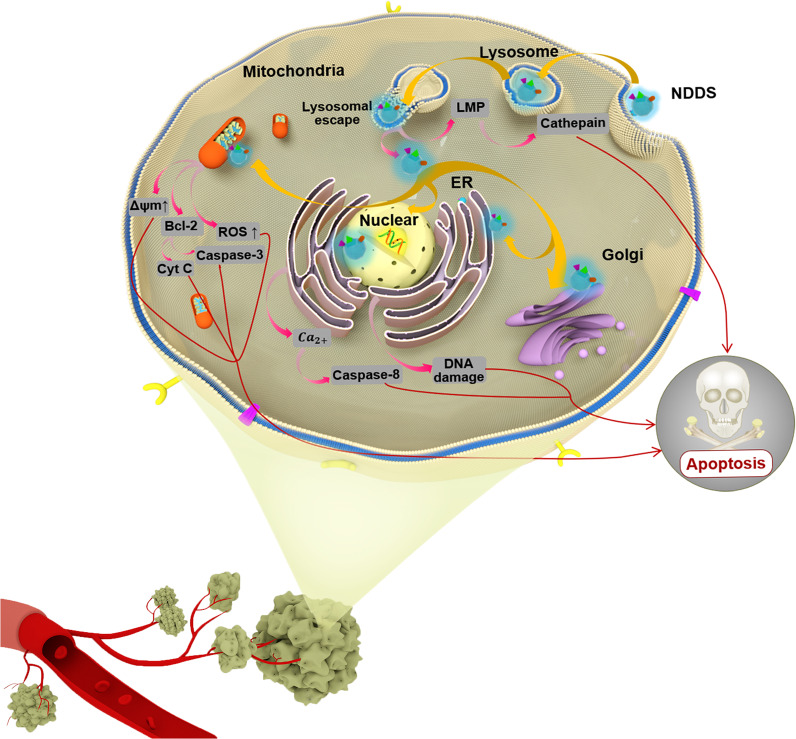
Schematic illustration of cancer cell subcellular targeting NDDSs for improving cancer therapeutic efficacy and different poptotic pathways mediated by different organelle-targeted NDDSs. NDDS nanoparticle-based drug delivery system, LMP lysosomal membrane permeabilization, ER endoplasmic reticulum, Bcl-3 B-cell lymphoma 3, ROS reactive oxygen species, Cyt C Cytochrome C

With the development of precision medicine, researchers have realized that variations in key intracellular biomolecules (genes and proteins), which are usually at the subcellular level, play a critical role in carcinogenesis and cancer development.^4–6^ Designing drug candidates based on molecular-level pathogenesis has become a new pattern and trend of drug discovery. For example, Ying et al. found that the expression level of sterol o-acyltransferase 1, which is responsible for transforming cholesterol into cholesterol ester-storage granules, is closely related to the poor prognosis of patients with liver cancer. Based on this, the research team proved that avasimibe, a small molecular inhibitor of sterol o-acyltransferase 1, had a good antitumor effect on patient-derived tumor tissue xenograft model of hepatocellular carcinoma, and provided new treatment strategies for tumor patients.^7^ Moreover, high-profile gene therapies also have to deliver the therapeutic genes into the cytoplasm or nucleus, where they can function. As a result, effective NDDSs should not only carry the therapeutic agents to the target tissues and cells but also deliver the drugs to distinct subcellular sites which mean organelles as targets accurately. They are considered to be one of the most promising approaches for cancer treatment. Through their proper design and specific modifications, subcellular-targeting nanoformulations are enriched in tumor cells, are internalized by endocytosis across the subcellular barriers (such as inner body embedding and lysosomal degradation)^8^ and target-specific subcellular structures (as shown in Fig. 1). This is then followed by the controlled release of therapeutic agents at the target sites, thus improving their antitumor efficacy, reducing their toxic and side effects, and overcoming the most critical limitation of intracellular drug delivery—MDR.^9^

In this review, based on the latest research progress over the past 5 years, we will focus on the important aspects of subcellular-targeting nanoformulations for cancer therapy. First, relevant knowledge including the specific endocytosis pathway of different nanoformulations taken up into cells and the pathological characteristics of tumor cell organelles are the key elements for guiding the construction of NDDSs, especially for the selection of targeting ligands. Next, according to the different subcellular targets of commonly used anticancer therapeutic strategies (chemical therapy, gene therapy, photodynamic therapy (PDT), etc.) applied after surgery, this article will elaborate on how to achieve precise subcellular targeting by functionalizing the surface of nanoparticles (NPs) with ligands and other means in the order of lysosome, nucleus, mitochondria, endoplasmic reticulum (ER), and Golgi apparatus. Furthermore, we will point out that multiple targeting and controlled release are crucial to the design and overall construction of the subcellular-targeting NDDSs. Finally, two challenges and potential directions to pursue in order to boost precise subcellular targeting are illustrated, which will benefit the transformation of NDDSs from laboratory research to clinical practice.

## Main

NDDSs can achieve the enrichment of tumor microenvironment, cell internalization, and intracellular delivery through passive or active targeting. In passive targeting, the size, shape, and surface charge of NPs can affect penetration and retention, thus significantly affecting their cell internalization and subcellular localization. For example, positively charged ultrasmall NPs have a higher affinity to the organelles such as mitochondria and nuclei, thereby promoting their intracellular permeability.^10^ Active targeting usually relies on the modification of localization group such as antibodies, ligands, etc., which have specific interaction with the receptor, thus leading to more significant effect than conventional treatment strategies. In intracellular transport and targeting, we still focus on these two aspects to explore design strategies of subcellular-targeting nanoformulations.

### Endocytosis and intracellular trafficking of nanoformulations

There are many targets (such as folate receptors, transferrin (Tf) receptors, antigens) which are usually overexpressed on the surface of cancer cells, and targeting them to maximize the drug accumulation around cancer cells have become a focus research to cancer therapy in recent decades. When NPs reach the cell surface through passive or active targeting, endocytosis is the main mechanism by which they are taken up by cancer cells. Different types of NDDSs rely on different cell endocytosis mechanisms to enter the cell, which ensures they internalize in specific intracellular regions.^11^ We will briefly review the classic endocytosis pathways for better prediction of the intracellular fate of nanoformulations.

**Fig. 2:**
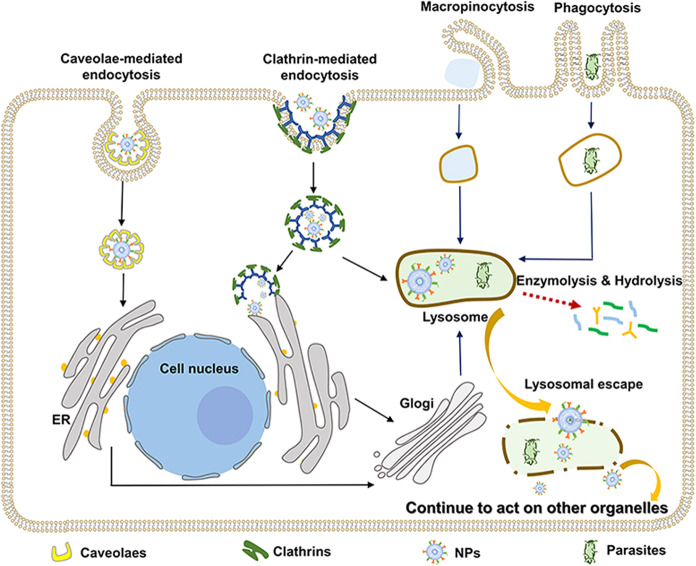
Schematic diagram depicting endocytosis and intracellular trafficking pathways of nanoformulations. NPs nanoparticles

Endocytosis can be divided into clathrin-mediated endocytosis (CME), caveolae-mediated endocytosis (CVME), macropinocytosis, and phagocytosis^12^ (as shown in Fig. 2). Among these, CME and CVME are the major uptake pathways of various nanoformulations. Generally, large NPs (<120 nm) are internalized mainly through CME, and specific ligand-modified nanoformulations (e.g., epidermal growth factor, folic acid, chemokines, and Tf) can significantly improve the efficiency of this endocytosis pathway. Following CME, the nanoformulations are trafficked through the early endosomes—late endosomes—lysosomes pathway and arrive in the lysosomal lumen, where they may be degraded by lysosomal hydrolases.^13^ For those nanoformulations whose action sites are other subcellular localizations in the cytoplasm, they are supposed to be designed to avoid endosome/lysosome degradation and retain their biological activity. Using carrier materials that are stable in acidic environments and solution with pH buffering properties can alleviate degradation problem to a certain extent.^14^ Endosome/lysosome escape capability is a more effective prerequisite.^15^ The commonly recognized mechanisms of lysosomal escape include proton-sponge effect, membrane fusion, the generation of gas, and the application of CPPs and PCI. Some examples and applications used in nanomedicine are listed in Table 1. On the other hand, nanoformulations with a small particle size (<60 nm) usually rely on CVME to enter cells. These NPs coated by caveolae usually do not enter lysosomes and are directly transferred to the Golgi or ER.^16,17^ Other endocytosis processes are shown in Fig. 2. As is apparent, the endocytosis process of antitumor NPs is the key step to achieve subcellular enrichment. Deep understanding and exploration of these endocytic pathways are rather significant for developing new delivery strategies for subcellular targeting.

**Table 1:** The mechanisms and applications of lysosomal escape used in nanomedicine

Mechanism of lysosomal escape	Device	The key structure for lysosomal escape	Cargoes	Cell line	Reference
Proton-sponge effect	catHDL/PA	Polyanions bearing both pendant carboxylate groups and alkyl chains	DOX and curcumin	T24	^117^
	N-quaternary ammonium-chitosan	Quaternary amine groups	Brucine	HepG2	^70^
	MPN-Coated NPs	The phenolic molecules in the metal–phenolic networks(MPNs)	Calcein	MDA-MB-231	^118^
	FL–C6-NH2-Modified CRP@dOSN	pH-responsive imine bonds	Chitosan	HeLa	^119^
	Polymeric-drug conjugate solid NPs containing encapsulated superparamagnetic iron oxide NPs (IO@PNP)	Poly(ethylene glycol)-block-poly(histidine)	DOX	PC3MM2	^120^
	Polyurethane micelles	Hydrazone bonds	DOX	SKOV3	^121^
	mPEG-b-PLA-PHis-ss-OEI	Disulfide bonds	DOX and siRNA	MCF-7/ADR	^122^
	Surface-modified single-walled carbon nanotube	Polyethylenimine (PEI)-betaine	DOX and siRNA	A549	^123^
	GA-loaded cp-rHDL NPs (cp-rHDL/GA)	The histidine in CPPs	Gambogic acid (GA)	HepG2 and HT1080	^124^
	Guanidino HPMA copolymer	Guanidine group	KLA peptide	B16F10	^125^
	TPH/PTX nanomicelles	The positively charged nanomicelles and HA	PTX	A549	^126^
	D-alpha-tocopheryl poly (ethylene glycol 1000) succinate and HA dual-functionalized cationic liposomes	The imidazole groups of histidine	PTX	MCF-7/MDR	^127^
	m-HA coating PEI-PCL/shRNA complexes	PEI-PCL	PTX and KIAA1199 specific shRNA	MDA-MB-231	^128^
	Dextran nanogels with CAD adjuvant	Cationic amphiphilic drugs adjuvant (nonbiodegradable polymeric dextran NGs, inorganic propylamine-functionalized MSNPs, cationic LNPs, such as (PEGylated) DOTAP-DOPE liposomes, the lipofection reagent Lipofectamine RNAiMAX, and lipid NPs containing the ionizable lipid DLin-MC3-DMA)	siRNA	H1299	^129^
	mPEG-PHis-PSD/PLL/siRNA NP	Poly(l-histidine)	siRNA	NSCLC	^130^
	Lipid NPs	The ionizable cationic lipid components	siRNA	HTB-177	^131^
	UCNP (CD/Azo) -siRNA/PEG NPs	GE11 + /TH + NP	siRNA	MDA-MB-468	^132^
	The cationic dextran nanogels	Cationic amphiphilic drugs	siRNA	H1299	^133^
	pH/redox dual-sensitive unimolecular NPs	The imidazole groups	siRNA	MDA-MB-468	^134^
	Poly(2-diethylaminoethyl methacrylate) around the silica nanoparticle core (PDEAEM@SNP)	The tertiary amine group of the PDEAEM shell	siRNA	MDA-MB-231	^14^
	siRNA biomimetic nanocomposites modified by erythrocyte membrane	Citraconic anhydride grafted poly-l-lysine	siRNA	U87MG	^135^
	DSPE-PEG-uPA@CaP	The CaP shell	siRNA and Pt	MDA-MB-231	^136^
	UA-GT/PAH-Cit/siRNA NCs	Imidazole-containing moieties	siVEGF	QGY-7703	^137^
	Angiopep LipoPCB(Temozolomide + BAP/siTGF-β)	The zwitterionic lipid (distearoyl phosphoethanol-amine-polycarboxybetaine lipid)	Temozolomide and siTGF-β	GL261	^138^
PCI	MSNs tethered with lipid bilayers (MSN@tLB)	IR-780	Zoledronic acid	MCF-7	^139^
	Photoactivatable Pt(IV) prodrug-backboned polymeric nanoparticle system (CNPPtCP/si(c-fos))	Azide complexes	Pt and si (c-fos)	A2780DDP	^140^
	Glucose functionalized polydopamine NPs	Polydopamine	Bortezomib borate	MDA-MB-231 and MCF-10A	^141^
	C60-DEX-NH2	Fullerenes	siRNA	MDA-MB-231 and 4T1	^142^
	The lipoic acid and chlorin e6‐conjugated pullulan micelle	Ce6	Chlorin e6 and DOX	HepG2, HeLa and HCT‐116	^143^
Membrane fusion	pBA‐CP‐pPEGA	The self‐assembly into tubisomes	\	HEK293	^144^
	Cross-linked N-(2-hydroxypropyl) methacrylamide copolymer micelles	HA2 peptide (GLFEAIEGFIENGWEGMIDGWYG)	H1-S6A and F8A (H1) peptide	MCF-7	^145^
CPPs	TAT-conjugated iron oxide NPs	TAT peptide	\	A549	^146^
	siRNA @ TMV-TAT	TAT peptide	siRNA	MHCC97-H/GFP	^147^
	siRNA /TAT-AP1-ELP complexes	TAT peptide	siRNA	4T1	^148^
	A hybrid nanoparticulate system based on a cationic helical polypeptide PPABLG (PPABDLG HNPs)	The helical structure of PPABLG features	siRNA	RAW 264.7	^149^
The generation of gas	siPol2 @ NPs	The chitosan-guanidinate-CO2 NPs	POLR2A siRNA	MDA-MB-453 TNBC	^150^
	D/N-PDA/Hb@HA	The NO donor	DOX	HeLa	^151^

### Lysosomal accumulation

Many nanoformulations mediated by CME can actively accumulate in lysosomes at the end of the endocytosis pathway. Taking full advantage of this accumulation to delivery antitumor drugs that act on lysosomes can greatly simplify the complexity of the carriers’ design. Second, recent reports have demonstrated chloroquine and its derivatives,^18^ rapamycin,^19^ HSP70 antagonist,^20^ and cathepsin B^21^ can act on the lysosomes and their components to trigger lysosomal membrane permeabilization (LMP), which can bypass the classical caspase apoptosis pathway and thus produce antitumor effects on drug-resistant cells.^22^ Third, the lysosomal pathological features lay a foundation for precise drug release.^23^ Given the evidence discussed above, lysosomal targeting and destruction could represent potential pharmacological delivery strategies.

#### Lysosomal characteristics

Lysosomes are single-membrane acidic vesicles (pH 4.5–5.0) that contain more than 60 hydrolytic enzymes that can break down biomolecules (such as proteins, lipids, carbohydrates, and nucleic acids).^24^ They play important roles in maintaining cellular homeostasis, inducing cell apoptosis, nutrient sensing, and immune responses.^22^ However, malignant transformation usually leads to changes in lysosomal volume, composition, and subcellular localization. In cancer cells, increased lysosomal fragility caused by increases in sphingomyelin makes lysosomes more vulnerable to LMP in response to stimuli, such as surfactants, heat, and reactive oxygen species (ROS), thus causing cell death.^25^

#### Delivery strategies of lysosomal precise therapy

Receptor-mediated endocytosis can usually increase the possibility of the NPs’ final arrival in lysosomes,^13^ so ligand modifications play important roles in lysosomal targeting. When NDDSs are modified by the specific aptamer of receptors on the surface of tumor cells, such as Tf^26^ and the anti-human epidermal growth factor receptor-2 monoclonal antibody,^27^ the receptor–ligand complex is mediated by receptor–ligand interactions, collected into transport vesicles and delivered into the early endosome-late endosome-lysosome pathway, resulting in its accumulation in lysosomes. Owen et al. reported NPs modified by different anti-HER2 mAbs (trastuzumab and 73JIgG) that bind to different epitopes on HER2 have variable amounts reaching the lysosome.^28^ Lysosome-targeting fragments can also be used to promote lysosomal accumulation. For example, alkylated piperidine fragments could target lysosomes and then self-assemble to construct anticancer prodrug molecules.^29^ In addition to surface modification, other physicochemical properties of NPs affect the efficiency of lysosomal accumulation. Lysosomal accumulation of internalized NPs is related to NP rigidity, size,^30^ and surface charge,^31^ and smaller and softer NPs with certain positive and negative charges have much greater uptake rates into lysosomes in cancer cells. Therefore, the main means of delivering drugs to lysosomes is to design and develop the appropriate targeting sequences, assisted by optimizing the physical and chemical properties of nanoformulations.

After reaching lysosomes, NDDSs need to respond to the lysosomal microenvironment effectively to release their cancer therapeutic agents, which need to act rapidly on the lysosome and trigger LMP. This response mainly relies on some pH-sensitive liposomes and stimulus-responsive polymers containing specific pH-triggered switches (such as disulfide bonds,^32^ hydrazone bonds, acrylic acid, and diethylaminophenyl units^33^) and enzyme response switches (such as cathepsin B-sensitive dipeptide linker,^34^ glycosidic bond hydrolyzed by glycosidase,^35^ vSIRPα-probe activated by lysosomal endopeptidases^36^). Additional important triggering methods are the delivery of photosensitizers^37^ and magnetic agents^24^ to lysosomes by NDDSs. When the tumor is exposed to external near-infrared light or a magnetic field, the sensitive agents will produce a considerable amount of ROS and heat, stimulating the destruction of the fragile lysosomal membrane, and induce tumor cell death. As shown by Zhang et al., their novel photosensitizer supramolecular nanogel is sensitive to lysosomal pH and aggregates in the lysosomes for enhanced PDT of multidrug-resistant cancer.^37^

### Nucleus targeting

Chemotherapy is still the cornerstone of cancer treatment and the vast majority of conventional chemotherapeutic drugs need to work in the nucleus of cancer cells to induce apoptosis.^38^ Alternatively, cancer gene therapy, which transfers genes (such as the CRISPR/Cas9 nuclease system, nucleic acid aptamers, DNA, and siRNA) to the chromosomes of tumor cells to regulate or replace abnormal genes, is gradually emerging.^39^ Their efficacy depends on the efficient transfer of the drugs or complete therapeutic exogenous gene into the nucleus.^40^ In recent studies, the nucleus has been commonly used as the site of action for free radicals and heat to cooperate with chemotherapy or gene therapy to improve the antitumor effect,^41^ which means transporting photosensitizers or theranostics to the nucleus to produce ROS with potentially damaging effects.^42,43^ However, the NDDSs targeting the cancer cell membrane generally only release foreign genes or anticancer agents into the cytoplasm, and then they can only enter the nucleus through free diffusion. The efficiency of diffusion is limited, and <1% of the therapeutic agents in the cytoplasm enter the nucleus and reach the final target.^38^ Therefore, enhanced therapeutic agent efficiency by nuclear targeted delivery is anticipated to be necessary for efficient cancer treatments and overcoming MDR.

#### Nuclear characteristics

The nucleus is the site of storage, replication, and transcription of genetic material and it plays important roles in cell proliferation, metabolism, growth, and differentiation. Due to the strong shielding effect of the bilayer nuclear membrane, nuclear pore complexes (NPCs) with lengths of ~90 nm and transverse diameters of 70 nm are the only channels for bidirectional exchange between the cytoplasm and nucleoplasm. The inner walls of NPCs are tethered with phenylalanine-glycine nucleoporins (FG Nups), thus limiting the inner diameter to only ~40 nm.^44^ As a result, the low efficiency of nuclear membrane penetration has greatly hindered applications of nuclear targeting NDDSs.

#### Construction strategies of cancer cell nucleus-targeting NDDSs

**Fig. 3:**
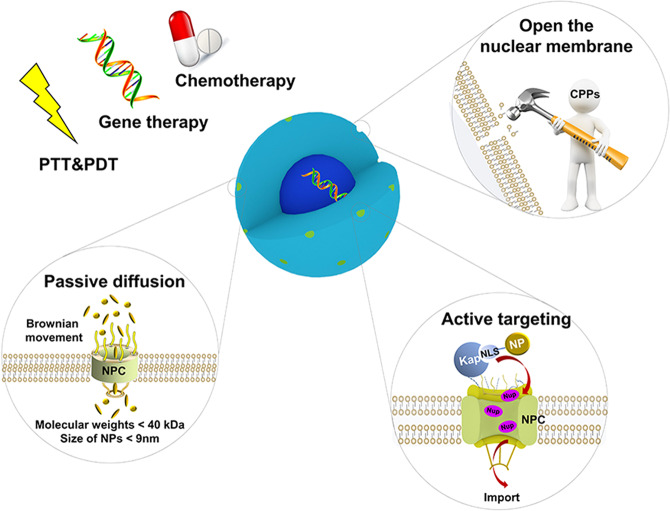
Three construction ways for nucleus-targeting NDDSs to access the cancer cell nucleus from the cytoplasm: passive diffusion, active targeting, and pore formation in the nuclear envelope membrane. PTT photothermal therapy, PDT photodynamic therapy, NPC nuclear pore complex, CPPs cell membrane penetrating peptides, Kap karyopherin, NLS nuclear localization signal sequence, NP nanoparticle

In general, the NDDSs’ ability to efficiently access the cancer cell nucleus from the cytoplasm arises from three aspects: passive diffusion, active targeting, and pore formation in the nuclear envelope membrane (as shown in Fig. 3).

##### Passive diffusion

The structure of the NPCs limits the translocation of nanoformulations into the nucleus by passive diffusion. Based on principles of Brownian motion, the key influencing factors of passive cancer cell nucleus-targeting NPs such as size, shape, and charge have been extensively studied as follows.

Size is the critical factor affecting the passive diffusion of NPs into the nucleus. Lim’s group has demonstrated that ions and small molecules with molecular weights <40 kDa can diffuse freely through the NPCs.^44^ For NDDSs, NPs capable of passive nuclear diffusion are generally smaller than 9 nm.^45^ Therefore, it is necessary for nucleus-targeting NPs to regulate their size by rational preparation or to achieve size reduction of large NPs activated by special pH conditions or enzymes.^46^ In particular, how to compress and fold gene macromolecules to minimize the size of the gene nanocarrier system should be considered. The existing research has mainly focused on how to condense DNA/RNA into stable complexes through the electrostatic interactions between cation nanocarriers and anion nucleic acids.^39^

Although small NPs are able to diffuse into the nucleus, the charge and shape of the NPs also play important roles in nuclear uptake. Positively charged NPs are more favorable for passage into the nucleus, but intravenous injection of positively charged NPs may induce hemolysis. To address this problem, a charge reversal strategy from negative to positive in endosomes and lysosomes has been applied.^47^ NDDSs that recover a positive charge in lysosomes can not only promote lysosomal escape but also enhance nuclear targeting, thus enhancing the cytotoxicity of the anticancer drug compared with free drugs.^48^ Other studies have shown that NPs with a higher aspect ratio (shaped like rods or worms) achieve higher nuclear concentrations compared with the lower aspect ratio NPs,^49^which can be ascribed to the structure of the NPCs.

##### Active targeting

Although the ultrasmall NPs can carry therapeutic agents into the cancer cells’ nucleus, most of the marketed NDDSs, whose sizes are usually between 100 and 200 nm, are excluded from the nucleus.^38^ Fortunately, NPs larger than NPC can realize nuclear active targeting by surface ligand modification after lysosomal escape.

Nuclear localization signal sequences (NLSs),^50^ including from the SV40 T antigen, adenovirus, transactivator of transcription (TAT) peptide, NF-κB, KRRRR et al.^51,52^ are the most classical ligands used for nuclear targeting. NLSs can be recognized by karyopherins (Kaps) and rapid binding between Kaps and FG Nups cause FG Nups to shrink back into more malleable forms.^53,54^ Therefore, NLSs modified NPs with a large particle size could enter the nucleus via active translocation. Thus far, most reported sizes of active nuclear targeting NDDSs were extended to 50 nm, which means gold NPs^55^ and mesoporous silica NPs (MSNs)^56^ have been extensively used in nucleus active targeting because of their advantages of easy control of particle size and surface modification. For example, Tang et al.^57^ synthesized copper sulfide NPs encapsulated by a silica shell layer, which were modified by RGD and TAT peptides at the same time. Mediated by RGD to enter cancer cells, these NPs can effectively target the nucleus with the help of TAT. When illuminated by a 980 nm laser, copper sulfide NPs release heat to rapidly increase the temperature and damage the DNA. Li et al.^58^ developed a kind of gold NPs with simultaneous surface modification of siRNA and NLSs. The NLS-mediated NPs translocated to the nucleus and the siRNA acted on gene promoter DNA methylation, thus inducing long-term gene silencing in the nucleus of cancer cells. Meanwhile, a promising strategy to transfer larger NPs to the nucleus involves optimizing the NLS density.^59^ For instance, compared to the high density of 2 NLS^2^/nm, NPs modified with the intermediate density of 0.9 NLS^2^/nm can achieve a 3.7-fold increased nuclear accumulation.^60^ In addition to NLSs ligands, boronic acid groups can also translocate anticancer NPs with a large size from the cell surface to the nucleus through the importin α/β-mediated pathway. In the future, the development and discovery of new NLSs will provide a wider range of options for targeted ligands of nuclear targeting NDDSs.

##### Opening the nuclear membrane

In addition to improving the physicochemical properties of the nanoformulations to pass through NPCs as readily as possible, another effective method is to open the nuclear membrane with the help of cell membrane penetrating peptides (CPPs)^61^ to enhance the nuclear translocation of antitumor NDDSs. Researchers have gradually mastered some common properties of CPPs and have synthesized a series of CPPs with stronger penetration and higher efficiency, such as CB5005,^62^ which consists of a membrane permeation sequence cascaded with the NF-κB NLS. Further study found this kind of CPP had a unique affinity to brain glioma and its application in adriamycin delivery could effectively penetrate the membranes of cancer cells and the nucleus, allowing the chemotherapy drugs to directly damage the DNA.

In short, nuclear delivery efficiency may depend on the physicochemical properties of the NPs including size, shape, charge, and surface modifications. Intensive study of these factors may allow for the development of efficient cell nucleus-targeting NDDSs. Meanwhile, besides light responses,^41^ further research is required to explore the means of controlling drug release from carriers in the nucleus.

### Mitochondria targeting

As indispensable energy reservoirs, mitochondria are also important as targets of anticancer drugs. Lonidamine, amlodipine, ceramide, and some natural substances (resveratrol, berberine, betulinic acid) are the main antitumor therapeutic agents acting on mitochondria. They usually activate the apoptotic effector proteins Bax and Bak, release cytochrome c, and form apoptotic bodies, inducing cancer cells’ death.^63,64^ Paclitaxel (PTX), doxorubicin (DOX), and camptothecin, in addition to acting on recognized targets, also act on mitochondria to varying degrees to induce apoptosis.^65^

Mitochondria in cancer cells show greater susceptibility than those in normal tissues. Thus, there is the potential to deliver radiosensitizers,^66^ photosensitizers,^67^ and theranostics^68^ to the mitochondria of cancer cells, aiming at ROS production and oxidative stress, which induce mitochondrial permeability transitions and fundamentally affect the energy supply of cancer cells. All of the above have demonstrated that mitochondria targeting is of great significance for improving antitumor therapy.

#### Mitochondrial characteristics

Mitochondria are double-membrane-bound organelles with independent DNA^69^ and they participate in multiple cellular functions, including energy production, calcium buffering, lipid synthesis, signaling, cell proliferation, and apoptosis.^70^ In the process of Adenosine triphosphate synthesis, protons are pumped from the mitochondrial matrix to the intermembrane space, which generates a proton gradient and establishes the mitochondrial membrane potential (MMP, about −160 mv).Cancer cells tend to experience mitochondrial dysfunction, such as an increased MMP (−220 mv), accumulation of hydrogen peroxide, reduced oxidative phosphorylation, increased ROS production, Ca^2+^ overload, and the Warburg effect,^71,72^ which mean that mitochondria in cancer cells are more susceptible to external disturbances than normal cells.

#### Construction strategies of cancer cell mitochondria-targeting NDDSs

**Fig. 4:**
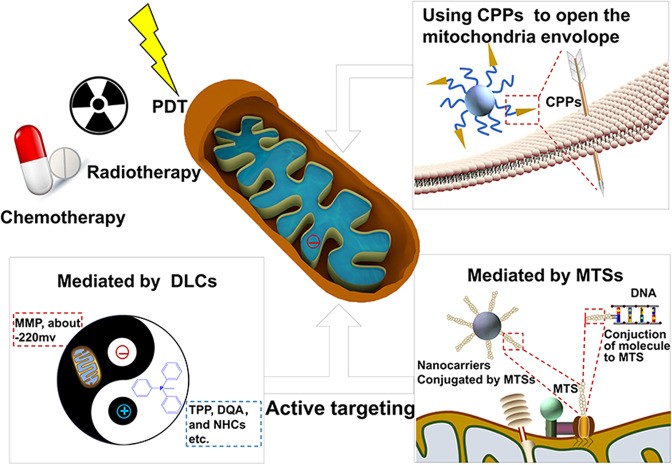
Schematic illustration of mitochondria-targeted drug delivery strategies mediated by active targeting and CPPs. PDT photodynamic therapy, DLCs delocalized lipophilic cations, MMP mitochondrial membrane potential, TPP triphenylphosphonium, DQA di-quaternary ammonium, NHCs nitrogen-containing heterocycles, CPPs cell membrane penetrating peptides, MTSs mitochondrial-targeting sequences

In view of the large negative MMP and the precise membrane structure of mitochondria in cancer cells, cancer cell mitochondria-targeting NDDSs usually achieve active subcellular targeting with the aid of two different targeting ligands: delocalized lipophilic cations (DLCs) and specific mitochondrial-targeting sequences (MTSs). Similarly, disturbing the mitochondrial membrane integrity by CPPs also helps NPs penetrate into mitochondria (as shown in Fig. 4).

##### Active targeting

DLCs, including 4-carboxybutyl triphenylphosphonium bromide (TPP), quaternary ammonium salts, nitrogen-containing heterocycles, et al.,^73^ can easily pass through lipid bilayers and accumulate in the mitochondrial matrix due to their high lipophilicity and stable cationic charge, so they have become the most popular constituent molecules in mitochondrial-targeting NDDSs.^74^ Among them, TPP has been the most extensively studied and it can both induce lysosomal escape and localize from the cytoplasm to the mitochondria. TPP-anchored poly(amidoamine) dendrimer,^75^ TPP-Lonidamine-DOX self-assembled NPs,^76^ PLGA-b-PEG NPs with surface modification of TPP,^77^ and silica NPs with surface modification of TPP^78^ can carry different therapeutic molecules to mitochondria in cancer cells. However, cationic materials represented by TPP induce inevitable systemic toxicity. Chemical modification of TPP or application of a core-shell structure, which shields the positive charges during circulation but is then removed in the lysosomal environment to expose the TPP, can maximize the safety of the drug. For instance, compared with liposomes where STPP is embedded in the lipid bilayer, liposomal loading with PTX and modification with a novel triphenyphosphonium-PEG-PE conjugate can more easily interact with the mitochondria and avoid the nonspecific cytotoxicity of STPP, to enhance their antitumor effects.^79^

In consideration of the safety concerns of DLCs, researchers have preferred to develop new MTSs to achieve precise intracellular localization. The precise membrane structure and internal structure of mitochondria provide a basis for determining the specific loci of MTSs. MTSs such as the KLA peptide^80^ and the amphipathic tail-anchoring peptide^81^ commonly contain 20–30 amino acids and α-helix structures that are rich in base, hydroxyl, and hydrophobic residues. Anticancer drugs, DNA, and nanocarriers conjugated to MTSs or DNA sequences encoding MTSs integrated into therapeutic DNA, are supposed to target mitochondria.^82^ For example, Kazuaki et al. designed a dual-function lipid-based drug delivery system that is capable of intracellular trafficking, such as endosomal escape mediated by octaarginine (a kind of CPP), and then delivery to mitochondria mediated by MTSs.^83^ However, it should be realized that extensive applications of MTSs are limited by their poor stability and their inability to target tumor locations. It is essential to improve the physicochemical and biopharmaceutical properties of these peptides and conjugate them with cancer cell-targeting fragments before clinical applications.

It is worth mentioning that the special mitochondrial microenvironment of cancer cells may be a trigger for drug release from vehicles. For example, Yue et al.^84^ reported the use of TL-CPT-PEG1K-TPP copolymers. After uptake by the cancer cells, the NPs were guided to the mitochondria by TPP, with hundreds of fold increased accumulation. The thioketal linker (TL) in the copolymers was sensitive to a high concentration of ROS in the cancer cells’ mitochondria to release CPT. Hu et al.^85^ constructed a kind of MSNs loaded with Fe^2+^. After mediated by the MTSs to enter the cancer cells’ mitochondria, the Fe^2+^ reacted with the increased amount of H_2_O_2_ and generated cell-damaging hydroxyl radicals. NIR exposure could promote chemical reactions and this delivery system has overcome the limitations that conventional PDT needs to rely on O_2_ and controllably exert their anticancer effects.

##### Opening the mitochondria envelope

Similar to the nuclear targeting delivery strategies, CPPs can also be used to enhance NDDSs penetration in accurate delivery of mitochondria targeting. Compared with the cell membrane, the mitochondrial membrane is more hydrophobic and has more negative potential, thus increasing the positive polarity and hydrophobicity of CPPs is conducive to helping nanoformulations cross the mitochondrial membrane. Commonly used mitochondrial CPPs generally have highly hydrophobic residues, such as cyclohexyl and SS peptides. In addition, some cationic small molecules, including rhodamine, pyridinium, and cyanine, which have inherent capabilities of mitochondrial penetration, can be modified on the surface of liposomes^86^ or self-assembled into NPs with imaging functions^87^ to selectively target the mitochondria.

In conclusion, an in-depth study of the cancer cells’ special mitochondrial microenvironment and development of novel targeting sequences may benefit the further design of efficient and safe mitochondrial-targeting NDDSs.

### ER and Golgi targeting nanoformulations

With the development of modern oncology, the discovery of new and valuable anticancer targets and cellular pathways has fostered the study of cancer therapeutic agents acting on organelles other than the nucleus and mitochondria. The ER and Golgi have gradually attracted attention due to their large intracellular surface area and important roles in endocytosis.

#### Characteristics of the ER and Golgi

As the largest subcellular structure in the cell, the ER is a series of lamellar and tubular cavities composed of membranes that are weakly alkaline and it stores large amounts of calcium. ER controls the biosynthesis, folding, and assembly of proteins and other biological macromolecules, as well as playing an important role in cell survival and homeostasis.^88^ When stimulated, the ER will release calcium ions and active caspase-8 to initiate the apoptotic program.^89^ Eeyarestatin, bortezomib, natural polyphenols, terpenes, and other ER stress inducers^90^ have been identified.^91^ Delivering anticancer drugs to cause sustained and excessive ER stress, thus inducing cell death, has become a new anticancer strategy.

The Golgi apparatus is closely linked to the ER. It is usually comprised of three different compartments, including the cis-Golgi network, medial-Golgi, and trans-Golgi network, which have a pH gradient from cis-Golgi network (pH 6.7) to trans-Golgi network (pH 6.0).^92^ It is an important organelle of cell secretory pathways that can modify, label, store and transport proteins, lipids, and polysaccharides. Recent studies have shown that the Golgi’s function is significantly improved in cancer cells, and its structural integrity affects certain signaling pathways, particularly those related to migration, invasion, and angiogenesis.^93,94^ Therefore, delivering intra-Golgi protein inhibitors to cancer cells’ Golgi has the potential to block multiple molecular pathways associated with the development of cancer.

#### Design of ER or Golgi targeting nanoformulations

In the delivery process of therapeutic agents acting on the ER or Golgi apparatus, it is necessary to consider the different endocytosis pathways of NPs entering cancer cells. That is, mainly because the CVME pathway can actively transport NPs into the ER and Golgi. Obviously, it is very beneficial to deliver antitumor agents to achieve CVME by specific design of their nanoformulations. The other key to designing NDDSs is to enhance their retention time in the target substructure and to avoid their being discharged by exocytosis. For example, Xue et al. reported a pH-responsive photothermal ablation agent that was assembled with bovine serum albumin to form NPs. Due to their hypertrophic morphology, they could accumulate in the Golgi apparatus of cancer cells during endocytosis. Meanwhile, NPs can be activated for effective photothermal therapy in response to the acidic environment of the Golgi.^95^

In addition, we must take into consideration that a significant proportion of NDDSs enter the lysosomes of cancer cells through the CME. For these NPs, only by modifying the appropriate ER and Golgi target sequences can they selectively target to these subcellular organelles after endolysosomal escape to the cytoplasm. Studies have shown that several biocompatible metal complexes could be used to target the ER. Kwon et al. reported an effective strategy for IR(III) delivery targeting the ER. The IR(III) complex can not only target the ER actively but also produce ROS in response to the PDT reagent, which results in oxidative damage to proteins.^96^ E3/19K of adenovirus,^97^ phosphotetrapeptide (4P),^98^ KKXX peptide,^99^ propylene oxide,^100^ the sulfonyl group,^101^ and ER-targeting photosensitizer TCPP-TER^102^ have also been applied to construct NPs targeting the ER.

In terms of Golgi targeting, Huang et al. demonstrated that L-cysteine is a kind of effective ligand for the Golgi. Carbon quantum dots and silica NPs could target the Golgi to monitor its changes when they are modified with l-cysteine.^103^ Gong’s team repeatedly proved that chondroitin sulfate (CS) nanomicelles targeted the Golgi since the glycosyltransferases in the Golgi could specifically bind to CS.^104,105^

However, it should be pointed out that compared with the targeting of the mitochondria and nuclei, the subcellular targeting of the ER and Golgi is still in its infancy, with not enough information available to apply comprehensive design strategies.

### Other subcellular-targeting nanoformulations

Apart from the above, there are several other important subcellular structures that are also susceptible to therapeutic agents.

The mutation of and abnormal expression of cytoskeleton-associated proteins play important roles in cancer cell migration, so targeting the cytoskeleton may be a potential anticancer therapy.^106^ For drugs (such as PTX and vincristine) acting on the cytoskeleton, the current delivery strategy is mainly to design NDDSs that are degraded in the lysosome, releasing the therapeutic agents into the cytoplasm through lysosome escape, and then achieve the drug targeting by the interaction between the drug molecules and the protein targets.^107^

As a complex of RNA and protein, many key molecules and proteins in ribosomes are secondary regulators of epigenetic regulation and cancer progression.^108,109^ In recent years, ribosomes have been gradually regarded as a potential target in the development of anticancer drugs.^110,111^ Discovery and delivery of drug molecules acting on ribosomes remains in a preliminary stage. Delivery of antitumor drugs to ribosomes will also be an important branch of subcellular targeting in the near future.

### Key factors in the rational design of subcellular-targeting anticancer nanomedicine

The above has described different strategies of precise delivery of antitumor agents to subcellular organelles in cancer cells. To guide the rational design and clinical transformation of subcellular-targeting anticancer nanomedicine comprehensively, we will emphasize below two key factors and principles that need to be considered when constructing efficient nanomedicine.

#### Dual targeting and multiple targeting

The initial premise of the subcellular-targeting NDDSs discussed above is that they have overcome the first step of initial delivery and tend to accumulate in the region of the tumor. Therefore, in the rational design of subcellular targeting anticancer nanomedicine, we need to use dual-targeting strategies, taking into account both cancer cell targeting and subcellular targeting. For example, Qu’s team^112^ designed folate and TAT-modified Fe_3_O_4_ core/mesoporous silica shell NPs to deliver camptothecin, López et al.^113^ developed mesoporous silica particles with asymmetric modification of folate and TPP, and Xie et al.^114^ constructed hollow carbonitride nanospheres modified by hyaluronic acid (HA) and mitochondrial localization peptide D. These NDDSs achieved the organic combination of cancer enrichment and subcellular level targeting, which greatly improving the efficiency of the antitumor agents. Furthermore, it should be noted that the overlapping interactions between two target ligands and their relative densities may have influences on their targeting ability. Meanwhile, scientists are also making efforts to synthesize multifunctional targeting sequences, such as one sequence having both cancer-targeting and subcellular targeting functions or having both navigation and imaging functions.

In many cases, targeting only one organelle may not be able to reach the expected therapeutic effect. One solution chosen by scientists is to simultaneously target multiple subcellular organelles or structures. For example, Yao et al.^115^ have developed HA-modified hydroxyapatite (HAP) NPs (HAP-HA). HA acts as a tumor-targeting active ligand and can bind to the CD44 receptor overexpressed on the surface of cancer cells. HAP can load and deliver DOX to the nucleus and mitochondria of tumor cells to maximize the expected therapeutic effect. Multiple targeting is based on the principle of organelle interaction network and functional synergy. Achieve simultaneous targeting of mitochondria and nucleus, ER and nucleus, as well as ER and mitochondria is of great significance for enhancing therapeutic efficacy.

#### Accurate response and controlled release

The differences between nanoformulations and free drugs lie not only in the protection and transport by the carriers but also in the controllable release of the cargoes in specific locations.^116^ Thus, subcellular-targeting nanoformulations are supposed to release their payload in a controlled manner to ensure that the goods cannot be released before reaching the specific target, but only be released on demand when they reach the target successfully. This response relies on the characteristics of the microenvironment in different organelles, such as the acidity of lysosomes,^32^ the weak basicity of the ER, the weak acidity of the Golgi,^95^ and the high expression of ROS^84^ and H_2_O_2_^85^ in the mitochondria. In-depth explorations of intracellular environments, components, and functionality will drive innovation in the development of promising subcellular-targeting NDDSs in the field of anticancer nanomedicine. It needs to be emphasized that in some programmed stimulus-response drug delivery systems, the use of two or more stimuli in sequential or coordinated action also requires comprehensive tests in vivo to achieve accurate spatiotemporal control of each trigger factor.

## Conclusions and perspectives

With the development of medical biology and nanotechnology, research into and applications of subcellular-targeting NDDSs have become hot topics and trends over the past 5 years. Great advances in nanotechnology have stimulated the quick development of various subcellular-targeting nanoformulations as listed in Table 2. They are generally modified with subcellular-targeting function groups to efficiently cross through the intracellular obstacles and reach the molecular target, where they control their payloads release in response to the specific subcellular microenvironment (e.g., the acidic environment of the lysosome and Golgi). This direct delivery of therapeutic agents to their final destination maximizes the therapeutic efficacy of various cancer therapies. Although progress in preclinical studies has been made, we have to point out that some limitations still remain. Here, we list the current challenges and potential future directions of this topic.

**Table 2:** Summary of the application of various subcellular-targeting nanoformulations in the past 5 years

Subcellular structures	Targeting molecules	Cargoes	Vehicles	Size and zeta	Cell lines	Others	Reference
ER	TCPP-TER	Porphyrin	Ds-sP/TCPP-TER NPs	100 nm	4T1 cells	PDT	^102^
	Fluorescent dansyl group	Tri-substituted triazine and 5-fluorouracil	A supramolecular self-assembled hexameric rosette structure	\	HeLa cells	\	^101^
	\	DOX	Ag NPs	75 nm	MCF-7/KCR cells	\	^152^
	Phosphoric acid tetrapeptide (1P)	Phosphoric acid tetrapeptide (1P)	The crescent-shaped supramolecular assemblies	\	HeLa cells	\	^98^
	\	Ir(III)	The Ir(III) complexes	\	HEK293T, U-2 OS or HeLa cells	PDT	^96^
	Adenovirus E3/19 K protein	The tumor-associated antigen L6	Cancer vaccine	\	EL4-L6 cells and B16F10 cells	\	^97^
Golgi	CS	DOX and retinoic acid	CS nanomicelles	40.2 ± 1.42 nm	Hepatic stellate cells	\	^104^
	CS	PTX and retinoic acid	CS nanomicelles	192.7 ± 1.8 nm	4T1-Luc cells	\	^105^
		Cyanine dyes	BSA-pH-PTT	\	HepG2 cells	PTT	^95^
	L-cysteine	Carbon quantum dots	Silica NPs	8.5 ± 3.5 nm	HEp-2 cells	\	^103^
Lysosome	Anti-HER2 mAb	\	Antibody drug conjugate polymeric NPs	\	BT-474 cells(HER2^+^ cell line)	\	^28^
	Anti-HER2 aptamer (human epidermal growth factor receptor-2, HApt)	\	Gold nanostars	90 nm, −8.05 mv	SK-BR-3 cells	\	^27^
	Tf	Dihydroartemisinin	Nanoscale Graphene oxide	100–200 nm	EMT6 cells	\	^26^
	EGF	\	Iron oxide magnetic NPs	14 ± 4 nm	MDA-MB-231 cells	Magnetic fluid hyperthermia	^24^
	\	Photosensitizer	Supramolecular nanogels and organosilica nanodots	75 nm	A549/DDP cells	\	^37^
	Alkylated piperidine fragment	Ferrocene analogs	N‐alkylamino ferrocene‐based prodrugs	\	BL-2 cells	The prodrug reacts with ROS.	^29^
	\	5,6-dimethylxanthenone-4-acetic acid	Direct-acting antiviral NPs	55 ± 2 nm	HeLa cells	PTT/PDT	^33^
Mitochondria	HA	Coumarin-6	HA/PEG/BD Nanodrugs	150 nm	A549 cells	\	^74^
	TPP	BSA, MAO-A, Cetuximab, IgG, or anti-MTCO2	CPD–TPP–protein@BS–NPs	\	HeLa, HepG2, and SH-SY5Y cells	\	^78^
	TPP	Lonidamine and DOX	TPP-LND-DOX NPs	110 nm	4T1, MCF-7, and MCF-7/ADR cells	\	^76^
	TPP	Lonidamine and α-tocopheryl succinate	poly(D,L-lactic-co-glycolic acid)-block (PLGA-b)-poly(ethylene glycol)-TPP polymer	\	HeLa, IMR-32, and 3T3-L1 cells	\	^77^
	TPP	α-tocopheryl succinate and obatoclax moieties	TOS-TPP-Obt-NPs	131.6 nm, 42.9 ± 1.20 mV	MDA-MB-231 cells	\	^153^
	TPP	Gd	TiO2(Gd) NPs	17.6 ± 0.1 mV	MCF-7 cells	Radiation therapy	^66^
	TPP	\	Poly(amidoamine) dendrimer	\	HeLa cells	\	^75^
	DSPE-PEG2K-TPP	Lonidamine and IR-780	Thermosensitive liposomes	125.0 ± 63.36 nm, 23.5 ± 3.12 mV	Lewis Lung Carcinoma cells	PTT/PDT	^68^
	TPP-PEG-PE	PTX	Liposome	145–175 nm,1.66 ± 5.49 mV	HeLa cells	\	^79^
	\	Fenton reagent	Upconversion NPs	\	HepG2 cells	\	^85^
Nuclear	Triplex-forming oligonucleotides	\	Tiopronin-covered gold NPs (Au-TIOP NPs)	<10 nm	MCF-7 cells	\	^45^
	Acridine based compounds	Chlorambucil	Acridin-9-methanol NPs	60 nm	HeLa cells	\	^154^
	AS1411 aptamers	Ce6	Ca-AS1411/Ce6/hemin@pHis-PEG (CACH-PEG) NCP	\	4T1 cells	PDT	^42^
	DGR or RGD, and KRRRR	Antisense single‐stranded DNA oligonucleotide	TD NCP/ASO-NPs	76–198 nm	MDA-MB-231 cells	Gene interference therapies	^52^
	FA and TAT	Camptothecin	MSNs	\	HeLa and A549 cells	Magnetic guidance	^112^
	H1 peptide	HA2	Cross-linked N-(2-hydroxypropyl) methacrylamide copolymer micelles	\	MCF-7 cells	\	^145^
	Membrane-permeable sequence (CB5005M)	DOX	\	\	Human glioma cells (U87)	Coordinately administered	^62^
	NLS	siRNA	Au NPs	\	MCF-7, HeLa, and HepG2 cells	\	^58^
	NLS	Iridium (III)	LNPdePEG-FA	150 nm	HeLa cells	\	^46^
	NLS	\	PPAP-DMA	150.6 ± 15.6 nm	HeLa cells	PDT	^43^
	NLS	Photosensitizer	Exosomes	132.6 nm	4T1 cells	PDT	^41^
	NLS	Albumin-Rhodamine	Chitosan NPs	150 nm	L929 cells	\	^59^
	RGD and NLS	\	Au NPs	\	HSC-3 cells	\	^55^
	TAT	DOX	MSNs	43 nm	MCF-7/ADR cells	\	^155^
	RGD and TAT	CuS	CuS@MSN-TAT-RGD NPs	40 nm, −23.9 ± 0.7 mV	HeLa cells	PTT	^57^
	TAT	DOX	MSNs	25/50/67/105 nm	HeLa cells	\	^56^
	TAT	DOX	NaYF4:Er/Yb@NaGdF4–PEG	58.8 nm	HeLa cells	\	^54^
	TAT	anti-p65 antibody and TAT peptide	MSNs	40 nm	4T1 cells	\	^156^
	HA and TAT	9-Nitro-20(S)-camptothecin	CHR–PCL–TAT–ALAL–HA (HATPC) micelles	121.6 ± 5.79 nm	SKOV3 tumor cells	\	^51^
Nuclear and mitochondria	HA and HAP	DOX	Hydroxyapatite NPs	179.50 ± 24.50 nm	HepG2 cells	\	^115^
	HA and KLA	DOX	Carbon nitride nanosphere	236.53 nm	A549 cells	\	^114^

In terms of cell biology research, the current progress related to the fate of subcellular-targeting nanomedicine may involve some uncertainties. (1) There is controversy since different researchers have come to different conclusions about the endocytosis pathway and mechanism of the same type of nanoformulation. (2) There is a lack of support from raw data and targeted research related to the stability of most currently existing nanoformulations in lysosomes, especially regarding how to ensure subcellular-targeting groups are able to function after escaping from the lysosome. (3) The intertumor heterogeneity is currently less considered in the design of subcellular-targeting NDDSs, which are mainly based on the common pathological features of organelles. Therefore, there is an urgent need for more comprehensive studies on different types of cancer cells (such as MDR cells) at the organelle/molecular level. In addition, precision medicine is based on gene mutation information, and individualized treatment, especially in subcellular delivery of gene therapeutic agents, should pay more attention to understanding the internal regulation of living systems by combining them with gene sequencing technology.

In terms of clinical transformation, the translation efficiency of complex nanoformulations is quite low. A high targeting ability of multiple modified structures is closely related to their instability, and a high sensitivity to intracellular environmental changes is often accompanied by systemic toxicity. This imbalance between efficacy and side effects makes demands on the exploration of multifunctional targeting groups (e.g., have both cancer-targeting and subcellular-targeting functions, have both navigation and imaging functions) on the one hand, and drives the development of diversification triggering and release strategies at the subcellular level (especially the nucleus) on the other hand. Furthermore, exploiting controllable preparation of nanoformulations in combination with other novel techniques such as microfluidic technology will control or optimize their properties more accurately.

In terms of monitoring methods, observing dynamic nanoformulations’ behavior in vivo and in tumor cells is indispensable to the biological and medical research of nanomedicine. However, the various visualization imaging techniques in the field of nanomedicine have their own advantages and disadvantages. For instance, the analysis conducted by transmission electron microscopy is static while having high resolution. Two-photon microscopy can observe tumor tissues directly in real time and in vivo, but it is limited by the imaging depth and the resolution at the subcellular level. Most of the organelle fluorescent dyes need to be used after cell membrane rupture and inactivation. To solve these problems will require complementation with numerous technologies on the basis of the existing tools, especially imaging methods for visualizing the actual process of nanoformulations entering single cancer cells. In addition, subcellular pharmacokinetics also affect the final efficacy of nanomedicine and should be paid more attention to, since it can be used for screening and transformation.

In general, subcellular-targeting NDDS are expected to play a greater role in cancer treatment and, where appropriate, of other diseases. It is also an inevitable trend in the field of personalized cancer medicine and precision nanomedicine. This review emphasizes the importance of subcellular targeting in the precise treatment of tumors, and encourages the development of novel subcellular-targeting strategies. The application of multidisciplinary and more concentrated efforts in the research into subcellular-targeting NDDSs and clinical transformation can further enhance our understanding of personalized cancer medicine for precise treatment and effectively guide the future design of nanoformulations.
